# 2-Meth­oxy-6-(6-methyl-1*H*-benzimidazol-2-yl)phenol

**DOI:** 10.1107/S1600536809016523

**Published:** 2009-05-14

**Authors:** Hui-Quan Xiao, Ming-Zhu Zhang, Wei Wang

**Affiliations:** a Department of Chemistry, Shaoxing University, Shaoxing 312000, People’s Republic of China; b College of Chemistry and Life Sciences, Zhejiang Normal University, Jinhua 321004, People’s Republic of China; c Yancheng Institute of Technology, School of Chemical and Biological Engineering, Yancheng 224003, People’s Republic of China

## Abstract

The mol­ecule of the title compound, C_15_H_14_N_2_O_2_ is almost planar, the dihedral angle between the 6-methyl-1*H*-benz­imidazole plane and the 2-methoxy­phenol plane being 6.9 (2)°. An intra­molecular O—H⋯N hydrogen bond is present. Adjacent mol­ecules are linked by N—H⋯O hydrogen bonds into a three-dimensional network structure. The benzoimidazole methyl group and its attached C atom are positionally disordered in a 0.724 (4):0.276 (4) ratio.

## Related literature

For background to imidazole and its derivatives, see: Huang *et al.* (2004 ▶) and to benzimidazoles, see: Perry & Wilson (1993 ▶). For related structures, see: Savall & Fontimayor (2008 ▶).
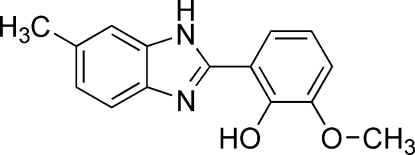


## Experimental

### 

#### Crystal data

C_15_H_14_N_2_O_2_
*M* *_r_* = 254.28
Monoclinic, 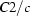

*a* = 17.986 (3) Å
*b* = 11.4452 (16) Å
*c* = 13.4105 (19) Å
β = 104.216 (2)°
*V* = 2676.1 (7) Å^3^
*Z* = 8
Mo *K*α radiation
μ = 0.09 mm^−1^
*T* = 293 K
0.21 × 0.17 × 0.13 mm

#### Data collection

Bruker APEXII CCD area-detector diffractometer
Absorption correction: multi-scan (*SADABS*; Sheldrick, 2003 ▶) *T*_min_ = 0.982, *T*_max_ = 0.989
6836 measured reflections
2531 independent reflections
1441 reflections with *I* > 2σ(*I*)
*R*_int_ = 0.031

#### Refinement

*R*[*F* ^2^ > 2σ(*F* ^2^)] = 0.056
*wR*(*F* ^2^) = 0.169
*S* = 1.05
2531 reflections
176 parameters
3 restraints
H-atom parameters constrained
Δρ_max_ = 0.20 e Å^−3^
Δρ_min_ = −0.23 e Å^−3^

### 

Data collection: *APEX2* (Bruker, 2004 ▶); cell refinement: *SAINT-Plus* (Bruker, 2001 ▶); data reduction: *SAINT-Plus*; program(s) used to solve structure: *SHELXS97* (Sheldrick, 2008 ▶); program(s) used to refine structure: *SHELXL97* (Sheldrick, 2008 ▶); molecular graphics: *SHELXTL* (Sheldrick, 2008 ▶); software used to prepare material for publication: *SHELXTL*.

## Supplementary Material

Crystal structure: contains datablocks I, global. DOI: 10.1107/S1600536809016523/hg2503sup1.cif

Structure factors: contains datablocks I. DOI: 10.1107/S1600536809016523/hg2503Isup2.hkl

Additional supplementary materials:  crystallographic information; 3D view; checkCIF report

## Figures and Tables

**Table 1:** Hydrogen-bond geometry (Å, °)

*D*—H⋯*A*	*D*—H	H⋯*A*	*D*⋯*A*	*D*—H⋯*A*
O1—H1⋯N2	0.82	1.83	2.567 (2)	148
N1—H1*A*⋯O2^i^	0.92	2.54	3.173 (3)	127
N1—H1*A*⋯O1^i^	0.92	2.06	2.920 (3)	155

Symmetry code: (i) 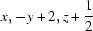.

**Fig. 1.**
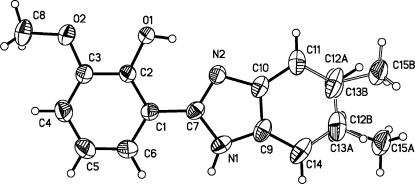
The independent molecules of the title compound, showing 30% probability displacement ellipsoids and the atom-numbering scheme.

## supplementary crystallographic information

### Comment

Imidazole and its derivatives are an important class of heterocycle with N-donor atoms, therefore they can be excellent organic ligands to generate various complexes (Huang *et al.*, 2004). Benzimidazoles are privileged structural units not only in the pharmaceutical industry but also in several other fields such as agricultural, electronic, and polymer chemistry (Perry *et al.*, 1993). We report here the synthesis and crystal structure of the title compound.

The molecular structure is shown in Fig. 1. The values of the geometric parameters in the compound are normal (Savall *et al.*, 2008) (Table 1). The benzimidazole and phenol groups are nearly coplanar, the dihedral angle between 6-methyl-1*H*-benzimidazole plane and 2-methoxyphenol plane is 6.9 (2)°. The compounds are linked by N—H···O hydrogen bonds [N1—H1A··· O1, N1—H1A···O2, O1—H1···N2] into a three-dimensional network structure.

### Experimental

A mixture of 4-methylbenzene-1,2-diamine (1 mmol) and 2-hydroxy-3-methoxybenzaldehyde (1 mmol) in ethanol (15 ml) was stirred for 8 h and then filtered. The resulting clear orange solution was vapor at room temperature for 7 d, after which orange block-shaped crystals of the title complex suitable for X-ray diffraction analysis were obtained, yield 45%.

### Refinement

The H atoms were fixed geometrically and were treated as riding on their parent C atoms, with C—H distances in the range of 0.93–0.96 Å and with *U*_iso_(H) = 1.2*U*_eq_(parent atom), or *U*_iso_(H) = 1.5*U*_eq_(C_methyl_). The coordinates of the H atoms of the N—H and O—H groups were found from difference Fourier maps and were allowed for as riding atoms with O—H 0.82 Å and N—H 0.92 Å and with *U*_iso_(H) = 1.2*U*_eq_(O).

### Crystal data

**Table:**

C_15_H_14_N_2_O_2_	*F*(000) = 1072
*M**_r_* = 254.28	*D*_x_ = 1.262 Mg m^−^^3^
Monoclinic, *C*2/*c*	Mo *K*α radiation, λ = 0.71073 Å
Hall symbol: -C 2yc	Cell parameters from 2318 reflections
*a* = 17.986 (3) Å	θ = 2.4–23.9°
*b* = 11.4452 (16) Å	µ = 0.09 mm^−^^1^
*c* = 13.4105 (19) Å	*T* = 293 K
β = 104.216 (2)°	Block, yellow
*V* = 2676.1 (7) Å^3^	0.21 × 0.17 × 0.13 mm
*Z* = 8	

### Data collection

**Table:**

Bruker APEXII CCD area-detector diffractometer	2531 independent reflections
Radiation source: fine-focus sealed tube	1441 reflections with *I* > 2σ(*I*)
graphite	*R*_int_ = 0.031
φ and ω scans	θ_max_ = 25.7°, θ_min_ = 2.1°
Absorption correction: multi-scan (*SADABS*; Sheldrick, 2003)	*h* = −21→11
*T*_min_ = 0.982, *T*_max_ = 0.989	*k* = −13→13
6836 measured reflections	*l* = −15→16

### Refinement

**Table:**

Refinement on *F*^2^	Primary atom site location: structure-invariant direct methods
Least-squares matrix: full	Secondary atom site location: difference Fourier map
*R*[*F*^2^ > 2σ(*F*^2^)] = 0.056	Hydrogen site location: inferred from neighbouring sites
*wR*(*F*^2^) = 0.169	H-atom parameters constrained
*S* = 1.05	*w* = 1/[σ^2^(*F*_o_^2^) + (0.0769*P*)^2^ + 0.8329*P*] where *P* = (*F*_o_^2^ + 2*F*_c_^2^)/3
2531 reflections	(Δ/σ)_max_ < 0.001
176 parameters	Δρ_max_ = 0.20 e Å^−^^3^
3 restraints	Δρ_min_ = −0.23 e Å^−^^3^

### Special details

**Table:**

Geometry. All e.s.d.'s (except the e.s.d. in the dihedral angle between two l.s. planes) are estimated using the full covariance matrix. The cell e.s.d.'s are taken into account individually in the estimation of e.s.d.'s in distances, angles and torsion angles; correlations between e.s.d.'s in cell parameters are only used when they are defined by crystal symmetry. An approximate (isotropic) treatment of cell e.s.d.'s is used for estimating e.s.d.'s involving l.s. planes.
Refinement. Refinement of *F*^2^ against ALL reflections. The weighted *R*-factor *wR* and goodness of fit *S* are based on *F*^2^, conventional *R*-factors *R* are based on *F*, with *F* set to zero for negative *F*^2^. The threshold expression of *F*^2^ > σ(*F*^2^) is used only for calculating *R*-factors(gt) *etc*. and is not relevant to the choice of reflections for refinement. *R*-factors based on *F*^2^ are statistically about twice as large as those based on *F*, and *R*- factors based on ALL data will be even larger.

### Fractional atomic coordinates and isotropic or equivalent isotropic displacement parameters (Å^2^)

**Table:**

	*x*	*y*	*z*	*U*_iso_*/*U*_eq_	Occ. (<1)
O1	0.34998 (10)	0.96117 (16)	0.14576 (12)	0.0718 (6)	
H1	0.3809	0.9222	0.1879	0.086*	
O2	0.23740 (12)	1.0753 (2)	0.03072 (14)	0.0942 (7)	
N1	0.39603 (12)	0.94335 (19)	0.46813 (14)	0.0644 (6)	
H1A	0.3689	0.9806	0.5087	0.077*	
N2	0.42667 (11)	0.89053 (17)	0.32288 (14)	0.0568 (5)	
C1	0.31924 (14)	1.0273 (2)	0.30233 (18)	0.0568 (6)	
C2	0.30615 (14)	1.0242 (2)	0.19534 (18)	0.0574 (6)	
C3	0.24529 (16)	1.0877 (2)	0.1342 (2)	0.0691 (8)	
C4	0.20037 (17)	1.1559 (3)	0.1795 (2)	0.0839 (9)	
H4	0.1605	1.1992	0.1389	0.101*	
C5	0.21404 (19)	1.1607 (3)	0.2856 (3)	0.0928 (10)	
H5	0.1834	1.2075	0.3158	0.111*	
C6	0.27225 (17)	1.0974 (3)	0.3463 (2)	0.0797 (9)	
H6	0.2807	1.1009	0.4175	0.096*	
C7	0.38041 (14)	0.9559 (2)	0.36368 (17)	0.0554 (6)	
C8	0.1738 (2)	1.1332 (4)	−0.0374 (3)	0.1217 (14)	
H8A	0.1748	1.1176	−0.1074	0.183*	
H8B	0.1776	1.2158	−0.0251	0.183*	
H8C	0.1266	1.1047	−0.0252	0.183*	
C9	0.45596 (15)	0.8650 (2)	0.49645 (19)	0.0659 (7)	
C10	0.47445 (14)	0.8322 (2)	0.40546 (19)	0.0618 (7)	
C11	0.53215 (16)	0.7501 (3)	0.4079 (2)	0.0806 (9)	
H11	0.5454	0.7270	0.3480	0.097*	
C14	0.49349 (18)	0.8175 (3)	0.5915 (2)	0.0865 (10)	
H14	0.4807	0.8398	0.6519	0.104*	
C12A	0.56835 (19)	0.7052 (3)	0.5008 (3)	0.1024 (16)	0.724 (4)
H12A	0.6072	0.6509	0.5033	0.123*	0.724 (4)
C13A	0.55061 (19)	0.7359 (3)	0.5920 (3)	0.0988 (17)	0.724 (4)
C15A	0.5957 (3)	0.6706 (5)	0.6876 (3)	0.1041 (15)	0.724 (4)
H15A	0.5797	0.6974	0.7469	0.156*	0.724 (4)
H15B	0.6495	0.6854	0.6969	0.156*	0.724 (4)
H15C	0.5861	0.5883	0.6788	0.156*	0.724 (4)
C13B	0.56835 (19)	0.7052 (3)	0.5008 (3)	0.0988 (17)	0.276 (4)
C12B	0.55061 (19)	0.7359 (3)	0.5920 (3)	0.1024 (16)	0.276 (4)
H12B	0.5769	0.7018	0.6536	0.123*	0.276 (4)
C15B	0.6306 (6)	0.6287 (11)	0.5397 (9)	0.1041 (15)	0.276 (4)
H15G	0.6498	0.5992	0.4838	0.156*	0.276 (4)
H15D	0.6136	0.5648	0.5748	0.156*	0.276 (4)
H15E	0.6707	0.6703	0.5868	0.156*	0.276 (4)

### Atomic displacement parameters (Å^2^)

**Table:**

	*U*^11^	*U*^22^	*U*^33^	*U*^12^	*U*^13^	*U*^23^
O1	0.0768 (12)	0.0922 (13)	0.0482 (10)	0.0351 (10)	0.0186 (9)	0.0116 (9)
O2	0.0935 (15)	0.1259 (18)	0.0566 (12)	0.0478 (13)	0.0059 (10)	0.0170 (11)
N1	0.0690 (14)	0.0807 (15)	0.0447 (12)	−0.0114 (12)	0.0164 (10)	0.0033 (11)
N2	0.0514 (12)	0.0654 (13)	0.0528 (12)	0.0031 (10)	0.0112 (10)	0.0091 (10)
C1	0.0562 (15)	0.0652 (15)	0.0518 (14)	0.0051 (13)	0.0186 (12)	0.0053 (12)
C2	0.0553 (14)	0.0654 (15)	0.0550 (15)	0.0130 (13)	0.0205 (12)	0.0091 (12)
C3	0.0693 (17)	0.0807 (19)	0.0588 (16)	0.0191 (15)	0.0185 (14)	0.0146 (14)
C4	0.0738 (19)	0.094 (2)	0.088 (2)	0.0316 (17)	0.0277 (17)	0.0206 (17)
C5	0.097 (2)	0.103 (2)	0.091 (2)	0.038 (2)	0.046 (2)	0.0105 (19)
C6	0.090 (2)	0.094 (2)	0.0645 (17)	0.0202 (18)	0.0352 (16)	0.0058 (15)
C7	0.0560 (14)	0.0641 (15)	0.0473 (14)	−0.0055 (13)	0.0147 (12)	0.0061 (11)
C8	0.116 (3)	0.151 (3)	0.080 (2)	0.059 (3)	−0.010 (2)	0.031 (2)
C9	0.0529 (15)	0.0782 (18)	0.0590 (16)	−0.0160 (14)	−0.0009 (13)	0.0180 (13)
C10	0.0528 (15)	0.0712 (17)	0.0565 (16)	−0.0081 (13)	0.0042 (12)	0.0166 (13)
C11	0.0611 (17)	0.086 (2)	0.087 (2)	0.0102 (16)	0.0036 (15)	0.0235 (16)
C14	0.079 (2)	0.112 (2)	0.0576 (17)	−0.0321 (19)	−0.0033 (15)	0.0269 (16)
C12A	0.080 (4)	0.103 (4)	0.116 (3)	0.015 (3)	0.006 (3)	0.042 (3)
C13A	0.070 (3)	0.129 (5)	0.077 (2)	−0.016 (4)	−0.020 (2)	0.055 (3)
C15A	0.101 (3)	0.131 (4)	0.072 (3)	0.028 (3)	0.005 (2)	0.034 (2)
C13B	0.070 (3)	0.129 (5)	0.077 (2)	−0.016 (4)	−0.020 (2)	0.055 (3)
C12B	0.080 (4)	0.103 (4)	0.116 (3)	0.015 (3)	0.006 (3)	0.042 (3)
C15B	0.101 (3)	0.131 (4)	0.072 (3)	0.028 (3)	0.005 (2)	0.034 (2)

### Geometric parameters (Å, °)

**Table:**

O1—C2	1.357 (3)	C8—H8A	0.9600
O1—H1	0.8200	C8—H8B	0.9600
O2—C3	1.367 (3)	C8—H8C	0.9600
O2—C8	1.438 (3)	C9—C10	1.393 (4)
N1—C7	1.367 (3)	C9—C14	1.397 (3)
N1—C9	1.382 (3)	C10—C11	1.394 (4)
N1—H1A	0.9194	C11—C12A	1.357 (4)
N2—C7	1.332 (3)	C11—H11	0.9300
N2—C10	1.394 (3)	C14—C13A	1.388 (5)
C1—C2	1.396 (3)	C14—H14	0.9300
C1—C6	1.397 (4)	C12A—C13A	1.383 (5)
C1—C7	1.453 (3)	C12A—H12A	0.9300
C2—C3	1.399 (3)	C13A—C15A	1.531 (4)
C3—C4	1.368 (4)	C15A—H15A	0.9600
C4—C5	1.384 (4)	C15A—H15B	0.9600
C4—H4	0.9300	C15A—H15C	0.9600
C5—C6	1.366 (4)	C15B—H15G	0.9600
C5—H5	0.9300	C15B—H15D	0.9600
C6—H6	0.9300	C15B—H15E	0.9600
			
C2—O1—H1	109.2	O2—C8—H8C	109.5
C3—O2—C8	117.7 (2)	H8A—C8—H8C	109.5
C7—N1—C9	107.4 (2)	H8B—C8—H8C	109.5
C7—N1—H1A	123.5	N1—C9—C10	105.8 (2)
C9—N1—H1A	129.0	N1—C9—C14	132.3 (3)
C7—N2—C10	105.6 (2)	C10—C9—C14	121.9 (3)
C2—C1—C6	118.8 (2)	C9—C10—C11	119.8 (2)
C2—C1—C7	118.8 (2)	C9—C10—N2	109.5 (2)
C6—C1—C7	122.5 (2)	C11—C10—N2	130.7 (3)
O1—C2—C1	123.0 (2)	C12A—C11—C10	117.6 (3)
O1—C2—C3	117.0 (2)	C12A—C11—H11	121.2
C1—C2—C3	120.0 (2)	C10—C11—H11	121.2
O2—C3—C4	125.8 (2)	C13A—C14—C9	117.3 (3)
O2—C3—C2	114.3 (2)	C13A—C14—H14	121.4
C4—C3—C2	119.9 (2)	C9—C14—H14	121.4
C3—C4—C5	120.3 (3)	C11—C12A—C13A	123.6 (3)
C3—C4—H4	119.9	C11—C12A—H12A	118.2
C5—C4—H4	119.9	C13A—C12A—H12A	118.2
C6—C5—C4	120.5 (3)	C12A—C13A—C14	119.8 (3)
C6—C5—H5	119.7	C12A—C13A—C15A	115.3 (4)
C4—C5—H5	119.7	C14—C13A—C15A	124.9 (4)
C5—C6—C1	120.6 (3)	C13A—C15A—H15A	109.5
C5—C6—H6	119.7	C13A—C15A—H15B	109.5
C1—C6—H6	119.7	H15A—C15A—H15B	109.5
N2—C7—N1	111.7 (2)	C13A—C15A—H15C	109.5
N2—C7—C1	123.1 (2)	H15A—C15A—H15C	109.5
N1—C7—C1	125.2 (2)	H15B—C15A—H15C	109.5
O2—C8—H8A	109.5	H15G—C15B—H15D	109.5
O2—C8—H8B	109.5	H15G—C15B—H15E	109.5
H8A—C8—H8B	109.5	H15D—C15B—H15E	109.5

### Hydrogen-bond geometry (Å, °)

**Table:**

*D*—H···*A*	*D*—H	H···*A*	*D*···*A*	*D*—H···*A*
O1—H1···N2	0.82	1.83	2.567 (2)	148
N1—H1A···O2^i^	0.92	2.54	3.173 (3)	127
N1—H1A···O1^i^	0.92	2.06	2.920 (3)	155

Symmetry codes: (i) *x*, −*y*+2, *z*+1/2.
